# Chromosome aberrations in canine multicentric lymphomas detected with comparative genomic hybridisation and a panel of single locus probes

**DOI:** 10.1038/sj.bjc.6601275

**Published:** 2003-10-14

**Authors:** R Thomas, K C Smith, E A Ostrander, F Galibert, M Breen

**Affiliations:** 1 Oncology Research Section, Animal Health Trust, Lanwades Park, Kentford, Newmarket, Suffolk CB8 7UU, UK; 2 Pathology Section, Animal Health Trust, Lanwades Park, Kentford, Newmarket, Suffolk CB8 7UU, UK; 3 Clinical Research and Human Biology Divisions, Fred Hutchinson Cancer Research Center, 1100 Fairview Ave. N. D4-100, PO Box 19024, Seattle, WA 98109-1024, USA; 4 UMR 6061 CNRS, Génétique et développement, Faculté de Médecine, 2 Avenue du Professeur Léon Bernard, 35043 Rennes Cédex, France; 5 Dept of Molecular Biomedical Sciences, College of Veterinary Medicine, North Carolina State University, Raleigh, NC 27606, USA

**Keywords:** canine, dog, lymphoma, chromosome, comparative genomic hybridisation (CGH)

## Abstract

Recurrent chromosome aberrations are frequently observed in human neoplastic cells and often correlate with other clinical and histopathological parameters of a given tumour type. The clinical presentation, histology and biology of many canine cancers closely parallels those of human malignancies. Since humans and dogs demonstrate extensive genome homology and share the same environment, it is expected that many canine cancers will also be associated with recurrent chromosome aberrations. To investigate this, we have performed molecular cytogenetic analyses on 25 cases of canine multicentric lymphoma. Comparative genomic hybridisation analysis demonstrated between one and 12 separate regions of chromosomal gain or loss within each case, involving 32 of the 38 canine autosomes. Genomic gains were almost twice as common as losses. Gain of dog chromosome (CFA) 13 was the most common aberration observed (12 of 25 cases), followed by gain of CFA 31 (eight cases) and loss of CFA 14 (five cases). Cytogenetic and histopathological data for each case are presented, and cytogenetic similarities with human non-Hodgkin's lymphoma are discussed. We have also assembled a panel of 41 canine chromosome-specific BAC probes that may be used for accurate and efficient chromosome identification in future studies of this nature.

Malignant lymphoma (lymphosarcoma) represents one of the most frequently encountered canine neoplasms, most commonly affecting middle-aged to older dogs of a wide range of breeds (Greenlee *et al*, 1990 and others). The disease originates from the malignant transformation of developing lymphocytes, and in the absence of chemotherapy, survival beyond one month after diagnosis is uncommon (MacEwan, 1990). Although generally considered a chemoresponsive form of malignancy in the dog, it is clear that, as with human non-Hodgkin's lymphoma (NHL), the disease is highly heterogeneous at both the clinical and histological level. A proportion of cases demonstrate more favourable response to therapy, and longer overall survival time, than others receiving the same initial diagnosis (Teske *et al*, 1994a).

A limited number of diagnostic and prognostic indicators have been described that may aid in the clinical management of canine lymphoma (e.g. Greenlee *et al*, 1990; Kuipel *et al*, 1999; Dobson *et al*, 2001), but it is evident that additional such aids are required in order to generate more refined modes of subclassification. Coupled with their physiological comparability, extensive genome homology, shared environment and the similarity in their clinical presentation of the disease, the domestic dog (*Canis familiaris*, CFA) represents an ideal system in which to study human multicentric lymphoma. As with many other human cancers, NHL is associated with a range of nonrandom chromosome aberrations (reviewed by Gaidano *et al*, 1993, Johansson *et al*, 1995; Sarris and Ford 1999 and others), a proportion of which has been shown to correlate with the clinical subtype of the disease. More recently, Alizadeh *et al* (2000) demonstrated the existence of marked variation in genome-wide gene expression profiles generated for human lymphoma patients who received a similar diagnosis. These factors represent potentially invaluable approaches to refining disease subclassification and assisting clinical diagnosis, prognosis and case management.

Since humans and dogs demonstrate extensive genome homology, it is likely that canine lymphoma will also be associated with recurrent chromosome aberrations. However, few reports exist describing chromosome abnormalities detected in canine lymphoma and at present insufficient data are available from which to draw significant conclusions on their findings. The most extensive study of this kind in the dog to date (Hahn *et al*, 1994) used conventional cytogenetic techniques to identify the single most commonly occurring aberration in chromosome preparations generated from each of 61 canine lymphoma cases. Of these, 70% demonstrated genomic imbalance as a consequence of aneuploidy (43 out of 61 cases), while balanced translocations were identified in the remaining 30% (18 out of 61 cases), identifying several potential trends that warrant further investigation. Studies of this kind, however, have been limited by difficulties in achieving accurate and consistent chromosome identification in the domestic dog, whose karyotype is notable for its high diploid chromosome number (2*n*=78), and the similar size, morphology and banding patterns of many of the autosomes, all of which are acrocentric. In addition, the reduced quality of chromosome preparations typically generated from tumour material further compound the practical limitations of a highly time-consuming purely classical approach.

The development of comparative genomic hybridisation (CGH) (Kallioniemi *et al*, 1992, 1994) as a technique for indirect analysis of chromosomal copy number changes in human tumour cells now provides a means by which imbalanced genomic aberrations can be identified accurately and efficiently without the need to generate tumour chromosome preparations. The application of CGH to the dog (Dunn *et al*, 2000; Thomas *et al*, 2001), the advent of novel molecular cytogenetic resources for this species (Langford *et al*, 1996), the development of comparative cytogenetic maps (Breen *et al*, 1999a, 2001; Yang *et al*, 1999; Sargan *et al*, 2000) and the generation of an integrated canine genome map (Breen *et al*, 2001) now provide a means by which to overcome prior practical difficulties and embark on more comprehensive studies of tumour karyotypes in this species. In addition to expanding our knowledge of the canine disease, the resulting comparative data will enable improved correlation between human and canine counterparts of a given cancer where they exist.

We have used CGH analysis to identify chromosome imbalances in 25 cases of canine multicentric lymphoma. The resulting range and distribution of aberrations observed indicates that, as with the human counterpart of this disease, the cytogenetic profiling of canine lymphoma as a potential aid to diagnosis and clinical management warrants more detailed investigation.

## MATERIALS AND METHODS

### Collection of case material

The 25 canine lymphoma cases described in this report were recruited from primary care veterinary practices throughout the UK during 1999–2001. Tumours represented suspected cases of multicentric malignant lymphoma on the basis of generalised lymphadenopathy, and the diagnosis was subsequently confirmed for each case by histological evaluation. The age at diagnosis ranged from 1 to 12 years (mean 8 years), and comprised 14 females and 11 males, of 12 different breeds (Table 1

**Table 1:** Summary of histopathological and cytogenetic data for 25 dog lymphoma cases

**Case no.**	**Sex/age**	**Breed**	**Classification (working formulation)**	**Classification (Kiel)**	**Immunophenotype/grade**	**Chromosome aberrations**
3971/01	♀/10	WHWT	Diffuse, small cell	Lymphocytic	B/low	+13
0882/01	♂/10	Border collie	Diffuse, small lymphocytic	Lymphoplasmacytic	B/low	+10/+27
3624/00	♀/6	X-breed	Diffuse, small lymphocytic	Lymphoplasmacytic	B/low	+1/−24qprox/−33/−36qprox
1354/00	♀/11	X-breed	Diffuse, mixed, small and large cell	Centroblastic-centrocytic	B/low	+31
3718/00	♂/5	Lurcher	Diffuse, mixed, small and large cell	Centroblastic-centrocytic	B/low	−14/+35
4033/00	♀/11	GSD	Diffuse, mixed, small and large cell	Centroblastic-centrocytic	B/low	−3qprox/+3qdist/−14
4757/00	♀/4	EBT	Diffuse, mixed, small and large cell	Centroblastic-centrocytic	B/low	+13/+31
1996/01	♂/10	Golden retriever	Diffuse, mixed, small and large cell	Centroblastic-centrocytic	B/low	+13/−14
4942/00	♂/7	Bull mastiff	Diffuse, mixed, small and large cell	Centroblastic-centrocytic	B/low	+13
4775/00	♂/8	X-breed	Diffuse, mixed, small and large cell	Centroblastic-centrocytic	T/low	−7qprox/+12/+13/−22/−23/+36/+38
4432/01	♀/6	Boxer	Diffuse, mixed, small and large cell	Centroblastic-centrocytic	T/low	+12/+13/+21qdist/+31/+36qdist/+37
1218/01	♀/9	Cocker spaniel	Diffuse, lymphoblastic	Lymphoblastic	B/high	−14/+27
3280/98	♂/9	X-breed	Diffuse, lymphoblastic	Lymphoblastic	B+T/high	−11/−30/+36/−38
4318/00	♂/8	Cocker spaniel	Diffuse, lymphoblastic	Lymphoblastic	T/high	+1/+6/−11/+20qprox/−28/+29/+30qprox/−30qdist/+31qdist/−34qdist/−38qdist
1947/01	♀/12	Cocker spaniel	Diffuse, lymphoblastic	Lymphoblastic	T/high	+4qprox-qmid/+6qprox/−8qprox/+9qdist/
						−11qdist/−16qdist/−19/−25/−33/+35/+36/−38
1935/99	♀/11	GSD	Diffuse, large cell	Centroblastic	B/high	+31
0233/00	♀/9	Boxer	Diffuse, large cell	Centroblastic	B/high	+31
2386/00	♂/9	X-breed	Diffuse, large cell	Centroblastic	B/high	+13/−22
4115/00	♀/9	Lurcher	Diffuse, large cell	Centroblastic	B/high	+13/+18
4517/00	♂/8	CKCP	Diffuse, large cell	Centroblastic	B/high	+31
0018/01	♂/1	Border collie	Diffuse, large cell	Centroblastic	B/high	+1/+13
1265/01	♀/8	Standard poodle	Diffuse, large cell	Centroblastic	B/high	+13/+16
1600/01	♀/6	Golden retriever	Diffuse, large cell	Centroblastic	B/high	−14
4290/01	♂/10	X-breed	Diffuse, large cell	Centroblastic	B/high	+13/+14qdist/+31
4641/00	♀/6	Bull mastiff	Diffuse, immunoblastic	Immunoblastic	B/high	+13

Case reference numbers are followed by the sex and age of the patient (in years), and the breed. Breed abbreviations are: West Highland white terrier (WHWT); German shepherd dog (GSD); English bull terrier (EBT); cavalier King Charles spaniel (CKCP). The classification of each case using both the Kiel and Working Formulation systems is then given, followed by the immunophenotype and grade of the case. The final column summarises the cytogenetic data for each case, and represents a brief list of the aberrations detected. The precise subchromosomal extent of each aberration is shown in Figure 3.

).

Following surgical excision, biopsied lymph node material was bisected and DNA was extracted from a representative region of tissue as described below. The remainder was collected into formalin for histological analysis. All biopsy procedures were performed prior to initiation of steroid treatment or chemotherapy.

### Histological and immunophenotyping analyses

Histological analysis and immunophenotyping of biopsied lymph node material was carried out using routine processing methods as previously described (Thomas *et al*, 2001), with the exception that tissue sections were immunolabelled using an indirect immunoperoxidase technique. Using the Techmate Horizon immunostaining apparatus (DAKO) according to the manufacturer's instructions, two sections were incubated in the primary antiserum for 1 h at room temperature. Anti-CD3 (pan T-cell marker) and anti-CD79a (pan B-cell marker) were diluted in DAKO antibody diluent to 1 : 400 and 1 : 100, respectively. Negative controls were generated by incubating two additional sections in diluent only, but mirrored all sequential steps of the two positive sections. Positive controls were provided by labelling sections of normal canine lymph node in parallel with the test sections. After washing for 10 min in DAKO buffer, sections were incubated for a further hour in rabbit/mouse EnVision (DAKO) conjugated to horseradish peroxidase. Tissue sections were then stained, mounted and examined as previously described (Thomas *et al*, 2001). Cases were classified by cell morphology and immunophenotype according to both the Working Formulation (National Cancer Institute, 1982) and the Kiel classification system (Lennert, 1978; Lennert and Feller, 1990).

### Tumour DNA extraction and probe labelling

Isolation of DNA from biopsied tumour material was performed as previously described (Thomas *et al*, 2001). Haematoxylin/eosin-stained sections were examined to establish that each specimen contained minimal (<10%) non-tumour tissue. DNA was also isolated from the peripheral blood lymphocytes of clinically normal male and female donors using the same method. Previous CGH analyses using DNA from these donors did not detect any statistically significant chromosome imbalances within either individual (Dunn *et al*, 2000). One microgram of tumour (test) and 1 *μ*g of normal (reference) DNA were labelled separately by incorporation of Spectrum Green- and Spectrum Red-dUTP (Vysis Illinois, USA), respectively, in a standard nick translation reaction in which the DNase I concentration was adjusted to result in the generation of fragments ranging from 100 to 1000 bp. The differentially labelled test and reference probes were then combined in the presence of canine C_o_t-1 competitor DNA, ethanol-precipitated and resuspended in hybridisation buffer as described by Dunn *et al* (2000).

### Image acquisition and CGH analysis

Fluorescence *in situ* hybridisation (FISH) and CGH analysis was performed as previously described (Dunn *et al*, 2000; Thomas *et al*, 2001). A minimum of 10 metaphase chromosome spreads, of comparable length and with minimal overlaps, were analysed individually using the QUIPS CGH analysis package (Vysis) to generate a relative copy number karyotype. Fluorescence profiles, representing the ratio of test : reference DNA, were plotted along the length of each chromosome against the corresponding DAPI-banded ideogram (Breen *et al*, 1999b). Profiles from individual metaphase spreads were then merged using the QUIPS CGH interpretation package (Vysis) to generate a mean fluorescence profile for each chromosome.

### Confirmation of chromosome identity

**Figure 1:**
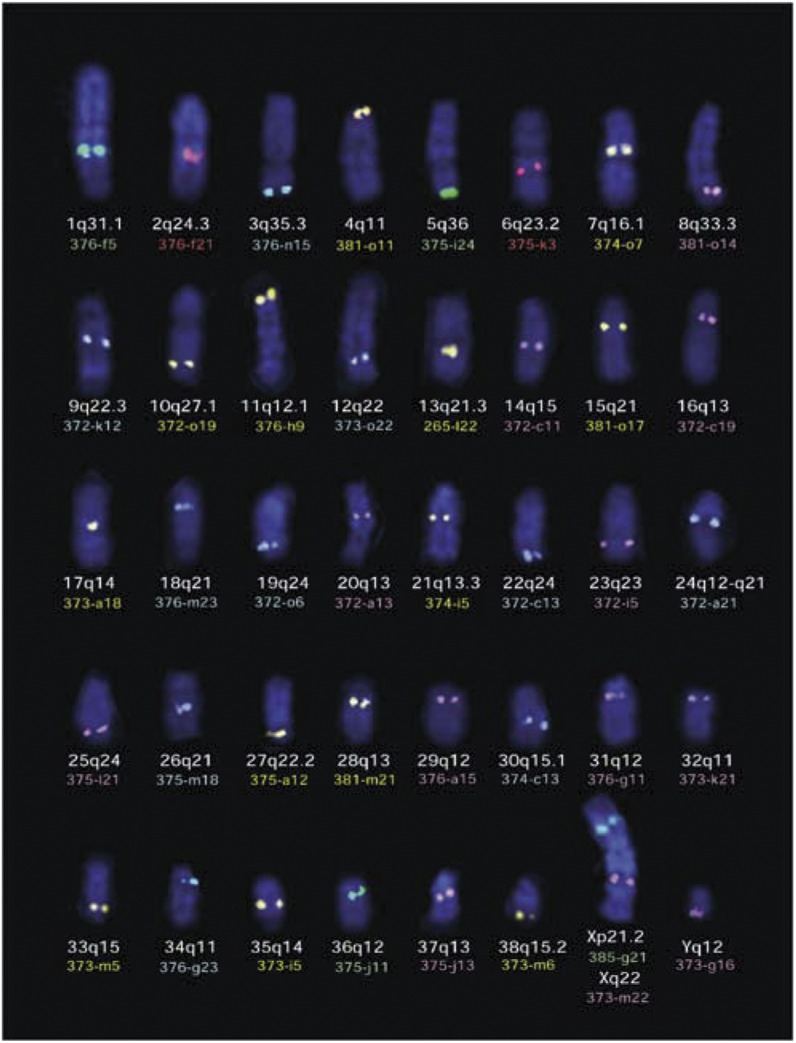
Cytogenetic distribution of 41 canine BAC clones representing a panel of chromosome-specific single locus FISH probes. In this example, probes are labelled with either Spectrum Red (presented as red signal), Spectrum Green (presented as green signal), Spectrum Gold (yellow), DEAC (blue) or biotin-Cy5 (pink). Each canine chromosome can be identified unequivocally using this panel of probes on the basis of the size of the chromosome, the cytogenetic location of the BAC clone and the fluorochrome with which it is labelled.

Chromosome imbalances detected by CGH analysis were identified by linear enhancement of the inverted DAPI component of each captured image, and comparison with the DAPI-banded dog karyotype of Breen *et al* (1999b). Where necessary, their identity and orientation was further verified using a panel of canine chromosome-specific single locus FISH probes (SLPs). This panel comprises 41 canine BAC clones, selected from the canine BAC library RPCI-81 (Li *et al*, 1999) on the basis of their cytogenetic position, as illustrated in Figure 1

. DNA was isolated from the panel of BAC clones using a standard alkaline lysis method, and 1 *μ*g of each was labelled for FISH analysis by standard nick translation to incorporate either diethylaminomethylcoumarin (DEAC)-5-dUTP (NEN), Spectrum Orange-dUTP (Vysis) or biotin-16-dUTP (Boehringer Mannheim, Germany). Use of these fluorochromes prevented spectral overlap with those used in CGH analysis. In compiling the panel of chromosome-specific SLPs, clones were selected on the basis of their cytogenetic location being in a centromeric, central or telomeric position (Figure 1). Consequently, the combination of fluorochrome and cytogenetic position allows multiple chromosomes to be identified in a single FISH reaction. Metaphase preparations analysed by CGH analysis were redenatured and exposed to additional rounds of FISH analysis using groups of differentially labelled SLPs (20 ng each) in the presence of 10 *μ*g of sonicated dog genomic DNA as competitor. Biotinylated probes were detected with Cy5-conjugated avidin (4 *μ*g ml^−1^, Amersham Pharmacia, Bucks, UK). Images for the same metaphase spreads acquired in the successive rounds of FISH analysis were compared with the original CGH image in order to confirm chromosome identity.

## RESULTS

### Histopathological evaluation and immunostaining

Of the 25 canine lymphoma cases analysed, 20 (80%) were of B-cell origin, and four (16%) were of T-cell origin. The remaining case demonstrated coexpression of both T- and B-cell antigens and has been described in detail elsewhere (Thomas *et al*, 2001). The panel comprised approximately equal numbers of low-grade (11 out of 25, 44%) and high-grade (14 out of 25, 56%) lymphomas. The predominance of B-cell lymphomas is broadly in line with previous reports, and with case numbers submitted for routine diagnostic evaluation from UK practices (data not shown), as is the slight predominance of high-grade over low-grade lymphomas. Six morphological subtypes were identified in total, with between one and nine representatives of each subtype.

### CGH analysis

**Figure 2:**
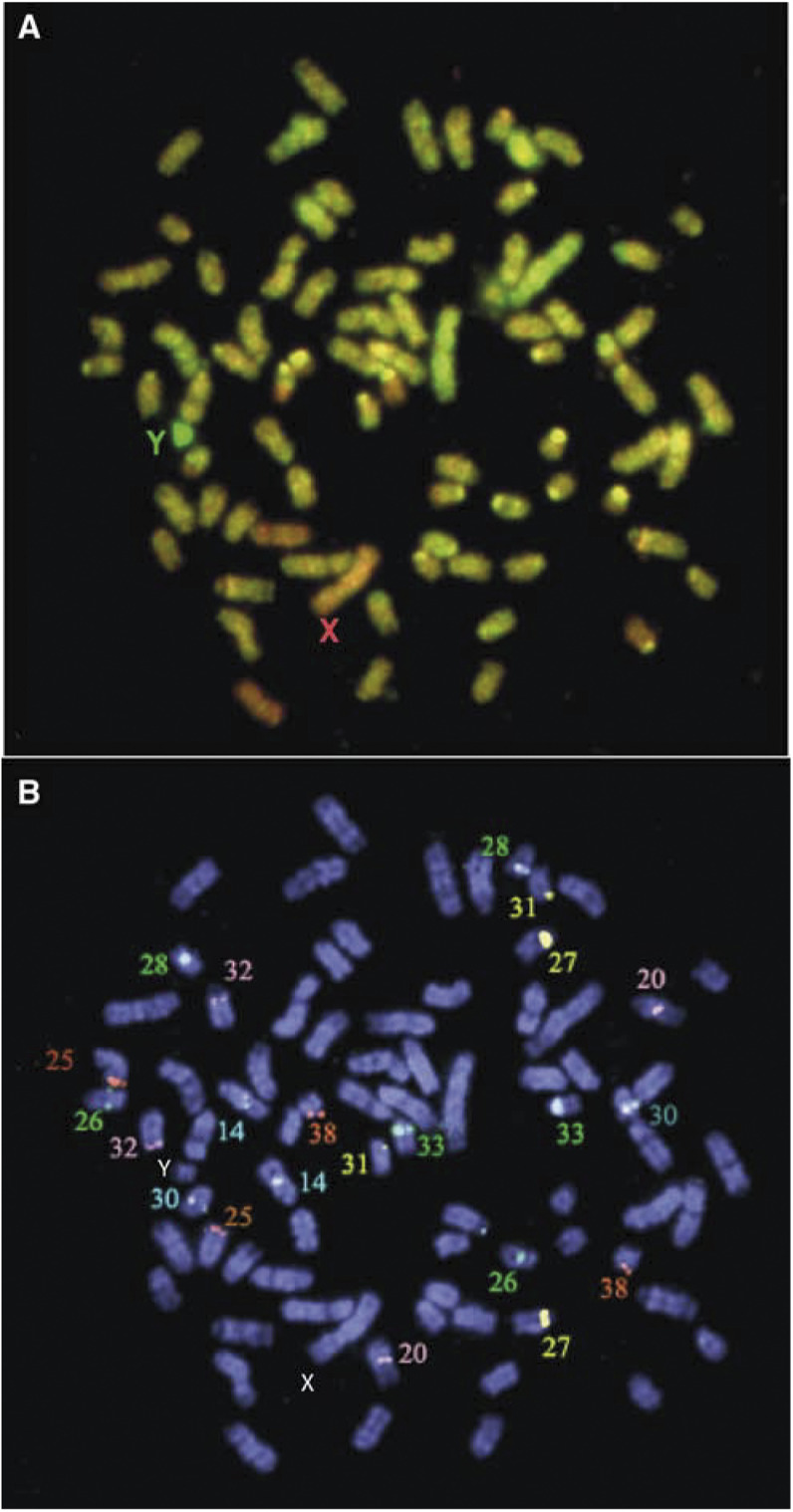
(**A**) Detection of unbalanced chromosome aberrations in canine lymphoma case 4138/00 using CGH analysis. Cohybridisation of male test (green) and female reference (red) probes onto a normal male metaphase spread is demonstrated. The sex-mismatch results in a 2 : 1 ratio of red : green for the X chromosome, which thus appears red. The Y chromosome appears green due to the 0 : 1 ratio of red:green for this chromosome. A number of autosomes appear to deviate from the expected 1 : 1 fluorochrome ratio. The complex microenvironment of the hybridisation reaction may produce apparent variation in hybridisation characteristics, as is evident in this cell for the CFA 31 homologues. However, profiling of 10–15 metaphase preparations serves to neutralise such differences. (**B**) Verification of chromosome identity using chromosome-specific single-locus probes. In this example, a total of 11 chromosome-specific single-locus probes were cohybridised in two successive reactions to the metaphase spread shown in (**A**) to facilitate accurate chromosome identification for CGH profiling. In the first reaction, the SLP for CFA 14 and CFA 30 were labelled with DEAC (presented as blue signal), CFA 26, CFA 28 and CFA 33 were labelled with Spectrum Orange (presented as green signal) and CFA 20 and CFA 32 were labelled with biotin-16-dUTP and detected with Cy5 (presented as pink signals). Following image acquisition, these probes were stripped from the chromosomes. The slides were then reprobed with a second group of SLPs, in which CFA 27 and CFA 31 were labelled in Spectrum Orange (presented as yellow signal) and CFA 25 and CFA 38 were labeled with DEAC (presented as orange signal). In this figure, the data from the two successive rounds of FISH have been overlaid to generate a composite metaphase spread showing all 11 SLPs.

An example of CGH analysis, and subsequent reprobing of the same metaphase for confirmation of chromosome identification, is presented in Figure 2

. Unbalanced chromosome aberrations were classed as those regions demonstrating a test : reference fluorescence ratio greater than 1.15 : 1 (gain) or less than 0.85 : 1 (loss), following standard conventions. Instances where a site of either gain or loss was interrupted by a short region falling marginally inside this range (and therefore not displaying statistically significant imbalance) were classed as a single aberration (i.e. gain of CFA 1 in case 0018/01; gain of CFA 6 in 4318/00; gain of CFA 13 in cases 1196/01, 4115/00 and 0018/01; loss of CFA 16 in 1947/01; loss of CFA 24 in 3624/00).

At least one unbalanced chromosome aberration was detected in each of the 25 cases studied, with a maximum of 12 aberrations detected in a single case (case 1947/01). In total, 76 aberrations were identified throughout the panel, equivalent to a mean of three per case. Of these, gains (48 out of 76, 63.8%) were significantly more common than losses (28 out of 76, 36.8%). Allowing for the convention of excluding centromeric and telomeric regions from CGH analysis, the majority of imbalances (59 of 76, 77.6%) were extensive, involving half or more of the physical length of the chromosome. Aberrations involved 32 of the 38 dog autosomes, with only CFA 2, 5, 15, 17, 26 and 32 demonstrating no detectable imbalance within any of the 25 lymphoma cases. Of these 32 autosomes, 16 (50%) showed only a single detectable instance of either over- or under-representation within the panel. The most common aberration detected was gain of CFA 13, present in 12 of the 25 cases (48%), followed by gain of CFA 31 in eight cases (32%). The most frequent losses were of CFA 14 (five of 25 cases, 20%), and of CFA 11 (three of 25 cases, 12%). It was most commonly observed that any given autosome demonstrated *either* gains *or* losses, with only six chromosomes (CFA 3, 14, 16, 30, 36 and 38) demonstrating at least one incidence of both over- and under-representation within the panel as a whole. In two instances (CFA 3 in case 4033/00; CFA 30 in case 4318/00), separate regions of both gain and loss were identified along the length of the same chromosome.

**Figure 3:**
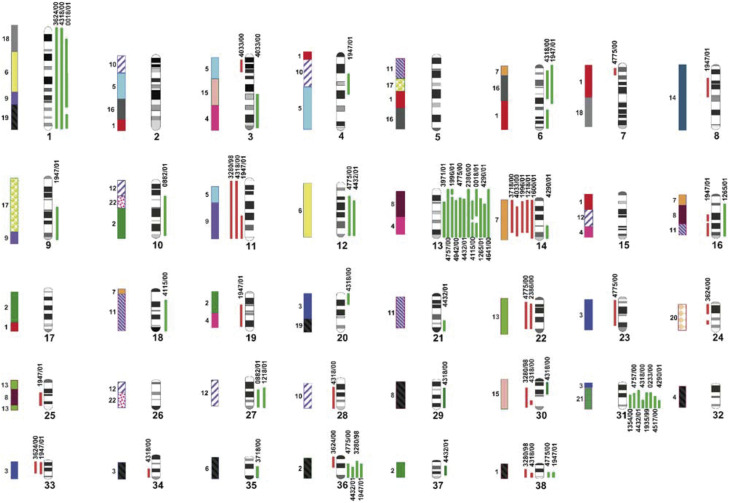
Composite of CGH profiles from 25 canine lymphoma cases. The DAPI-banded ideogram of Breen *et al* (1999a) is displayed. For each case, genomic gains and losses are shown as green and red bars to the right and left of each chromosome, respectively. Each vertical bar represents a site of genomic imbalance in a single case (cases are identified at the top or bottom of red/green bars), and demonstrates the physical extent of the chromosome over which the aberration was detected. The evolutionarily conserved chromosome segments shared with the human karyotype (taken from Breen *et al*, 2001) are identified with coloured bars to the far left of each chromosome.

Patient details, and histopathological and cytogenetic data are summarised in Table 1. Figure 3

presents the compiled CGH data obtained for each of the 25 cases analysed, showing the precise physical extent of each region of imbalance.

## DISCUSSION

CGH analysis of the 25 canine lymphomas demonstrated a total of 76 instances of significant genomic imbalance. The most striking trend observed was the gain of CFA 13 in almost half of the cases studied (12 of 25, 48%). This correlates with the findings of Hahn *et al* (1994), who reported trisomy of CFA 13 as the most common unbalanced aberration identified in their panel of cases studied by conventional cytogenetics (15 of 61 cases, 24.6%). Interestingly, the authors suggested a direct correlation between the existence of trisomy CFA 13 and an increase in both first remission length and survival time. In the present study, gain of CFA 13 was found in four of the six morphological subgroups of lymphoma identified, being absent in both lymphoplasmacytic and lymphoblastic lymphomas (see Table 1). Comparative mapping studies using reciprocal chromosome painting (Breen *et al*, 1999a; Yang *et al*, 1999) and radiation-hybrid mapping (Breen *et al*, 2001) show that CFA 13 is evolutionarily related to sites on two human chromosomes, HSA 8q23-qtel and HSA 4pprox-qprox. The former of these human chromosome sites harbours the *c-myc* oncogene, and the latter the *c-KIT* oncogene. Activation of human *c-myc* has been shown to be a common event in intermediate- and high-grade NHL (Gaidano *et al*, 1993) and so the recurrent gain of CFA 13 observed in this study suggests that it is crucial to examine the potential involvement of *c-MYC* and *c-KIT* in canine tumour development.

Of the other common aberrations observed in this study, gain of CFA 31 (eight of 25 cases) was largely restricted to centroblastic and centroblastic–centrocytic lymphomas. CFA 31 shares conserved synteny with HSA 21 (Breen *et al*, 1999a; Yang *et al*, 1999). Although aberrations of HSA 21 do not appear to be common in human lymphoma, HSA 21 amplification is frequently in a number of other cancers (e.g. Cortes *et al*, 1995; Perkins *et al*, 1997; Loncarevic *et al*, 1999). The overabundance of genetic material associated with disorders resulting from trisomy 21 has also been widely discussed. This may prompt wider investigation of the coding sequences shared by HSA 21 and CFA 31 and their significance in disorders resulting from gene dosage abnormalities. Loss of CFA 11 was identified solely in three of the four lymphoblastic lymphomas, and represents genetic material shared with HSA 5 and HSA 9 (Breen *et al*, 1999a; Yang *et al*, 1999). Although these human chromosomes do not appear to represent regions commonly aberrant in human lymphoma, it is important to consider that species-specific abnormalities may also play a role and must be evaluated with equal interest.

In addition to the potential correlation between *c-MYC* and the frequent gain of CFA 13 in canine lymphoma, the present report identifies several other possible evolutionary counterparts of aberrations found in human NHL. Gain of CFA 1 was noted in three cases within the present study, including a region evolutionarily related to HSA 18qdist. Monni *et al* (1996, 1997) demonstrated, using CGH analysis, that this human chromosome region is commonly amplified in several NHL subtypes and can be directly correlated with aberrant overexpression of a specific oncogene, *BCL2* (HSA 18q21.3), contained within the amplified chromosome region (HSA 18q21–q23). Gain of material from CFA 35 in two cases may correspond to the common amplification of HSA 6p in human lymphoma (e.g Monni *et al*, 1996) as these sites share an evolutionary origin (Breen *et al*, 1999a; Yang *et al*, 1999). Clearly, cytogenetic analysis of a greater sample size, supported by genomic sequencing and gene expression studies, will be required to establish whether the observed trends are significant, as well as the degree to which human and dog lymphoma share a common genetic aetiology.

A mean number of three chromosome aberrations was detected in each case. Four cases are notable for a marked increase above the mean, demonstrating between six and 12 aberrations each. It is interesting to observe that these represent the four T-cell lymphomas within the panel of 25 cases. This finding correlates with previous reports of a higher overall frequency of secondary chromosome aberrations in human T-cell lymphomas than those of B-cell origin, with both subtypes appearing to show nonrandom distribution (Johansson *et al*, 1995). It is also widely suggested that B-cell lymphomas typically demonstrate a more favourable prognosis than T-cell lymphomas. Similarly, Teske *et al* (1994a) and others have indicated a tendency towards an extended survival time and improved overall prognosis in canine B-cell lymphomas as compared to those of T-cell origin. The challenge for molecular cytogenetics is in part to establish additional means by which to subclassify existing broad categories of B- and T-cell lymphoma in the dog, and ultimately to correlate cytogenetic profiles with clinical progression and prognosis. We are therefore now pursuing a larger sample size to determine whether this would continue to support the tendency of B-cell lymphomas towards fewer chromosome imbalances, whether subtype-specific aberrations do exist, and in turn whether the total number of aberrations detected in each canine lymphoma case can be correlated with overall prognosis.

Studies of chromosome aberrations in other dog cancers may also provide support for the significance of specific chromosome regions in tumorigenesis. As with lymphoma, such studies have been limited by the poor quality of banding of tumour chromosome preparations, but certain features can be noted in common with the present report. Gain of CFA 13 has been reported in a dog lipoma (Reimann *et al*, 1999), and gain of CFA 1 in a case of acute leukaemia (Reimann *et al*, 1998) and of mammary tumour (Mayr *et al*, 1996). Gain of both CFA 1 and CFA 13 has also been demonstrated in a canine glial tumour (Dunn *et al*, 2000). Currently, the number of individuals and of cancer types studied in each case is insufficient for the assessment of recurrent aberrations and their significance.

The efficiency of CGH analysis in the domestic dog currently remains limited by the challenges of efficient and conclusive chromosome identification throughout the karyotype. Similarly, although prior studies of karyotypic aberrations in canine lymphoma (and other cancers) exist, it is difficult to determine the degree to which they correlate with the findings presented here, due to the absence of definitive chromosome identification in previous studies, and the use of different chromosome nomenclatures. In order to maintain consistency in (and correlation between) future reports, we therefore support the continued use of the standard karyotype nomenclature (Breen *et al*, 1999b) endorsed by the canine genome mapping community in the most recent integrated genome map (Breen *et al*, 2001). We also propose the use of the panel of 41 canine single-locus BAC probes described in this study as an accurate, efficient and universally available resource for the verification of chromosome identity. This resource will be key to the continued evaluation of karyotypic abnormalities in canine malignancies, whether by CGH analysis or by direct analysis of tumour metaphases.

In order to both complement and expand upon the findings of the present study, the identification of balanced aberrations by direct cytogenetic evaluation of tumour karyotypes will be an essential requirement for future work. This will rely heavily upon the ability to obtain viable tumour material and also to overcome previously documented limitations in generating high-quality chromosome preparations from biopsied tumour tissue (e.g. Carter *et al*, 1990; Hahn *et al*, 1994 and others).

A number of limitations are recognised within the present study. In common with our previous reports of canine CGH analysis (Dunn *et al*, 2000; Thomas *et al*, 2001), we observed intense and consistent hybridisation signals of both test and reference probes at the centromeres of a subset of dog chromosomes, reflecting suboptimal suppression of highly repetitive DNA sequences. Following conventions in human CGH (e.g. Speicher *et al*, 1993), we elected to exclude such sites from analysis. Similarly, we observed a site of apparently natural polymorphism between unrelated individuals within CFA 9qprox (Thomas *et al*, 2001), which was again excluded due to the risk of detecting false-positive genome imbalance (Kallioniemi *et al*, 1994). No other such regions were identified. Although all biopsy procedures were made at the time of initial diagnosis, it is not possible from this study to establish which aberrations represent primary changes involved in tumour initiation, and which are secondary changes involved in further progression of the tumour. Sequential sampling and cytogenetic analysis of a panel of lymphomas from the onset of hyperplasia onwards would help to elucidate which are the primary aberrations, but clearly has practical limitations.

The requirements of any CGH study include the need to generate high-quality metaphase preparations, the ability to identify each chromosome reliably, the resolution of metaphase-based technology and the time required to perform the analysis. We are currently developing a canine CGH genomic microarray, based on the assembly of a collection of ordered BAC clones that are dispersed throughout the canine genome at an average interval of 5 Mb. This resource will enable canine CGH analysis to proceed at a much faster rate and with greater resolution than is currently possible using conventional methods. Array-based techniques also enable such studies to be undertaken by laboratories without prior cytogenetics experience.

Limitations also exist with histopathological analyses of canine lymphoma, including the absence of a standardised system for morphological subclassification. We have therefore classified each case described in the present study using the two most commonly utilised schemes, enabling ongoing comparison of cytogenetic data with other parameters discussed in previous studies. As is common in the histological evaluation of canine lymphoma for routine diagnosis, immunophenotyping was restricted to the use of CD3 and CD79a cell markers. The application of more comprehensive immunostaining panels for canine lymphoma, as practiced in humans, may be of value in parallel with cytogenetics in refining disease classification (reviewed in Mason and Gatter, 1998). The imminent introduction of the new WHO classification for canine lymphoma (Parodi, 2001) as an evolution of the REAL system used in human (Frizzera, 1997) may be advantageous in that respect, through the incorporation of immunophenotyping data in classification, in addition to simplification of the morphological subtypes recognised. Retrospective and prospective analysis of lymphoma series with the newer classification systems will be necessary to establish their worth in the dog, and to establish the degree of comparability between canine and human lymphoma.

This study highlights the recurring nature of cytogenetic aberrations associated with canine lymphoma and suggests several trends, both within the canine disease and in comparison with its human counterpart, that are clearly worthy of continued investigation. It now becomes imperative to develop means by which to perform similar analyses on a greater sample size, and to integrate these findings with those from direct cytogenetic evaluation of tumour karyotypes. This combined approach will enable us to determine definitively the relative frequency and identity of both balanced and unbalanced chromosome aberrations present in canine lymphoma, and to investigate their clinical relevance. By establishing the degree to which these cytogenetic abnormalities correlate with the human counterpart of the disease, we may also establish how readily diagnostic and prognostic indicators identified in one species may be applied to the other, and may ultimately lead to a greater understanding of the precise genetic events involved in tumour initiation and development.
